# Myosin 1c: A novel regulator of glucose uptake in brown adipocytes

**DOI:** 10.1016/j.molmet.2021.101247

**Published:** 2021-05-07

**Authors:** Alice Åslund, Muhammad Hamza Bokhari, Erika Wetterdal, René Martin, Hans-Joachim Knölker, Tore Bengtsson

**Affiliations:** 1 Department of Molecular Biosciences, Wenner-Gren Institute, Stockholm University, SE-106 91, Stockholm, Sweden; 2 Faculty of Chemistry, Technical University of Dresden, Bergstrasse 66, 01069, Dresden, Germany

**Keywords:** Brown adipose tissue, Myosin 1c, GLUT1, PKA, p38, ATF-2

## Abstract

**Objective:**

The potential of brown adipose tissue (BAT) to influence energy homeostasis in animals and humans is encouraging as this tissue can increase fatty acid and glucose utilization to produce heat through uncoupling protein 1 (UCP1), but the actual mechanism of how the cell regulates glucose uptake is not fully understood. Myosin 1c (Myo1c) is an unconventional motor protein involved in several cellular processes, including insulin-mediated glucose uptake via GLUT4 vesicle fusion in white adipocytes, but its role in glucose uptake in BAT has not previously been investigated.

**Methods:**

Using the specific inhibitor pentachloropseudilin (PClP), a neutralizing antibody assay, and siRNA, we examined the role of Myo1c in mechanisms leading to glucose uptake both in vitro in isolated mouse primary adipocytes and in vivo in mice.

**Results:**

Our results show that inhibition of Myo1c removes insulin-stimulated glucose uptake in white adipocytes, while inducing glucose uptake in brown adipocytes, independent of GLUT4, by increasing the expression, translation, and translocation of GLUT1 to the plasma membrane. Inhibition of Myo1c leads to the activation of PKA and downstream substrates p38 and ATF-2, which are known to be involved in the expression of β-adrenergic genes.

**Conclusions:**

Myo1c is a PKA repressor and regulates glucose uptake into BAT.

## Highlights

• Myo1c is a BAT-specific regulator of glucose uptake.
• Myo1c inhibition leads to increased expression, translation, and translocation of GLUT1.
• Myo1c inhibition results in increased activation of PKA and its downstream targets.

## Graphical abstract

**Figure:**
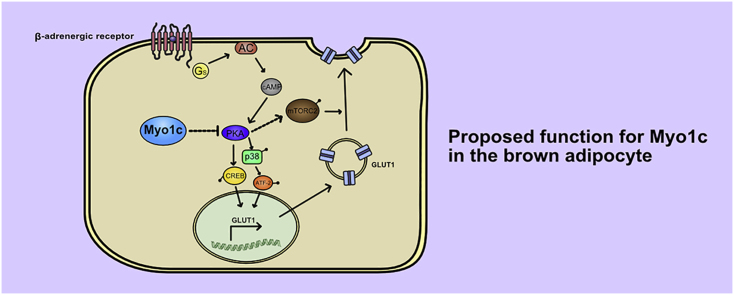

## 1 Introduction

In contrast to white adipose tissue (WAT), whose main function is to store energy as triglycerides in depots, brown adipose tissue (BAT) is activated by release of norepinephrine (NE) by sympathetic nerve endings to initiate catabolic and anabolic signaling cascades downstream of second messenger cyclic adenosine monophosphate (cAMP) to engage in non-shivering thermogenesis [1]. Despite its small volume, BAT has a high capacity for increasing glucose uptake and fatty acid consumption [1] impacting whole-body energy homeostasis, which makes it an attractive target to treat metabolic diseases. Whereas insulin-stimulated glucose uptake in BAT is regulated via glucose transporter 4 (GLUT4) translocation to the plasma membrane, β-adrenergic stimulation utilizes glucose transporter 1 (GLUT1) as the major mediator to uptake glucose [[2], [3], [4], [5], [6]]. We previously showed that β-AR-mediated glucose uptake to BAT in rodents occurs primarily through the β_3_-AR, involving an increase in cAMP levels and activation of mammalian target of rapamycin complex 2 (mTORC2), resulting in both de novo synthesis and translocation of GLUT1 to the plasma membrane independent of phosphatidylinositol 3 kinase (PI3K), Akt activation, and UCP1 expression [4,7,8]. However, many factors concerning the glucose uptake mechanism in BAT are still unknown.

As it was previously reported that reorganization of the cytoskeleton [9] or addition of latrunculin B, an agent that disrupts the actin cytoskeleton [10], inhibits β_3_-AR stimulated glucose uptake [4], we aimed to further investigate potential proteins involved in actin rearrangement leading to glucose uptake in BAT. Unconventional myosins are a family of motor proteins that interact with actin to initiate several cellular key processes mediated through ATP hydrolysis. Myosin 1c (Myo1c) is expressed in a wide range of tissues with enrichment near the plasma membrane. It has been shown to be important for multiple functions related to actin architecture, membrane dynamics, cell adhesion, regulation of the mitotic spindle, and gene transcription [11]. It is a ∼118 kDa monomeric protein that contains three domains: a motor domain that hydrolyzes ATP and binds to actin, a neck domain that contains regulatory sites, and a tail domain that interacts with cargo and other proteins via a pleckstrin homology (PH) domain and localizes to the plasma membrane as well as the cell nucleus and lipid membranes throughout the cell [[12], [13], [14]]. It has also been shown to be involved in both insulin-and contractile-mediated glucose uptake and is required for proper GLUT4 vesicle fusion in white adipocytes and skeletal muscle [[15], [16], [17], [18]]. Although expressed in BAT [19], its function has, to the best of our knowledge, not been previously investigated in brown adipocytes.

In this study, we investigated the role of Myo1c regarding insulin- and β-adrenergic-stimulated glucose uptake in vitro into primary white and brown adipocytes and basal uptake into adipose tissue in vivo. For this purpose, we used pentachloropseudilin (PClP), a highly specific, reversible, and allosteric inhibitor that selectively targets the ATPase and motor activity of Myo1c in mammalian cells [20,21] together with a neutralizing antibody assay and siRNA to study the specific effects on glucose uptake by inhibition of Myo1c. We show for the first time that Myo1c inhibition results in glucose uptake into BAT, but not WAT, as a consequence of increased GLUT1 expression, translation, and translocation.

## 2 Results

### 2.1 Myosin 1c inhibition removes glucose uptake in white adipocytes, but induces glucose uptake in brown adipocytes and BAT

**Figure 1:**
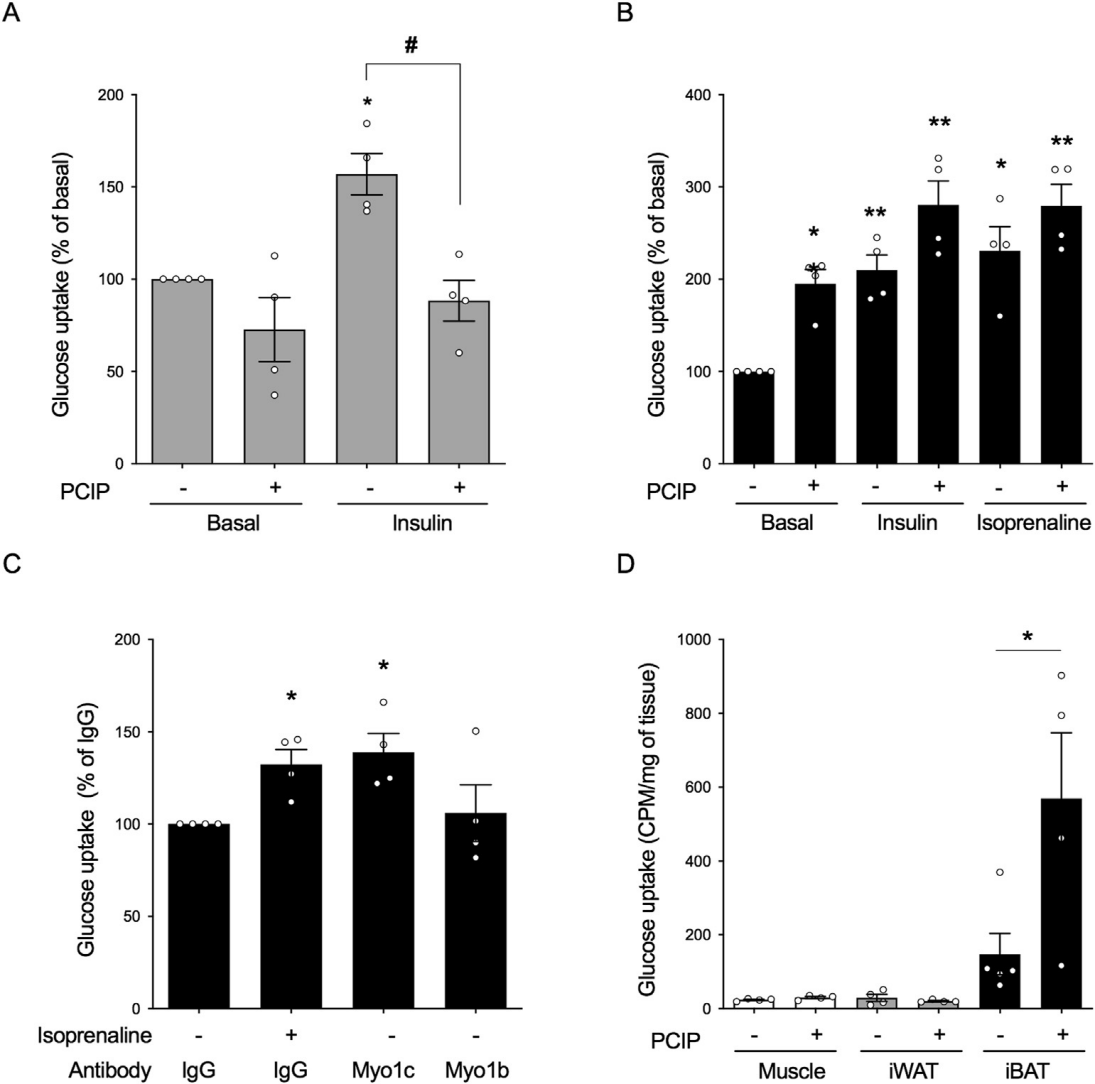
**Myosin 1c inhibition induces glucose uptake exclusively in BAT**. (A) The effect of 5 μM of PClP, a Myo1c inhibitor, on insulin (1 μM)-stimulated glucose uptake in white adipocytes normalized to basal. Cells were pretreated with PClP for 30 min prior to stimulation with insulin for 2 h (n = 3). (B) The effect of 5 μM of PClP on glucose uptake in brown adipocytes in response to insulin (1 μM) and isoprenaline (1 μM) normalized to basal. Cells were pretreated with PClP for 30 min prior to stimulation with insulin and isoprenaline for 5 h (n = 4). (C) Glucose uptake in brown adipocytes with isoprenaline (1 μM) and in response to antibodies neutralizing Myo1c and Myo1b for 8-10 h normalized to IgG (n = 4). (D) In vivo glucose uptake in BAT, WAT, and gastrocnemius in C57Bl/6N mice treated with PClP (5 mg/kg body weight i.p.) or vehicle (DMSO) for 2.5 h (n = 4 mice). Each value represents mean ± SEM. Statistics for (C) were calculated using one-way ANOVA with Dunn's multiple comparisons post-test. Statistics for the remaining data presented were calculated using two-way ANOVA followed by Tukey's multiple comparisons post-test. ∗P < 0.05 and ∗∗P < 0.01 compared to controls. # represents statistical differences within groups for P < 0.05.

To investigate the effect of Myo1c inhibition on insulin-induced glucose uptake in white adipocytes as previously reported for 3T3-L1-cells [15,16], cells were treated with PClP (5 μM) 30 min before being stimulated with insulin (1 μM) for 2 h (Figure 1A). To induce glucose uptake with a full adrenergic response in brown adipocytes coupled with de novo GLUT1 translation and translocation, the cells were treated with the agonists for 5 h (Figure 1B). While PClP fully inhibited insulin-mediated glucose uptake in white adipocytes (Figure 1A), interestingly, it failed to do so in brown adipocytes (Figure 1B). Similarly, β-adrenergic stimulation by isoprenaline (1 μM) for 5 h was not affected by the inhibitor (Figure 1B). Remarkably, a significant increase in basal glucose uptake could be observed in brown adipocytes treated with the inhibitor alone (Figure 1B). When Myo1c function was inhibited by administration of its corresponding antibody into live cells for 10 h, glucose uptake was significantly induced compared to the negative control IgG and myosin 1b (Myo1b), which demonstrated the specificity of isoform Myo1c to this event (Figure 1C). PClP treatment for various durations (up to 5 h) and concentrations (5 mM) did not affect adipocyte morphology or the cytoskeleton or result in either cellular stress or apoptotic activation (data not shown). We examined the effect of Myo1c inhibition by PClP on glucose uptake in vivo with mice treated with PClP (5 mg/kg) or vehicle for 2.5 h. In mice treated with the inhibitor, there was a significant increase in glucose uptake in BAT compared to the controls. Of note, this uptake was absent in both muscle and WAT (Figure 1D). Collectively, these results indicate a clear difference in tissue response between BAT, WAT, and muscle.

### 2.2 Myosin 1c inhibition induces glucose uptake through increased transcription, de novo synthesis, and translocation of GLUT1

**Figure 2:**
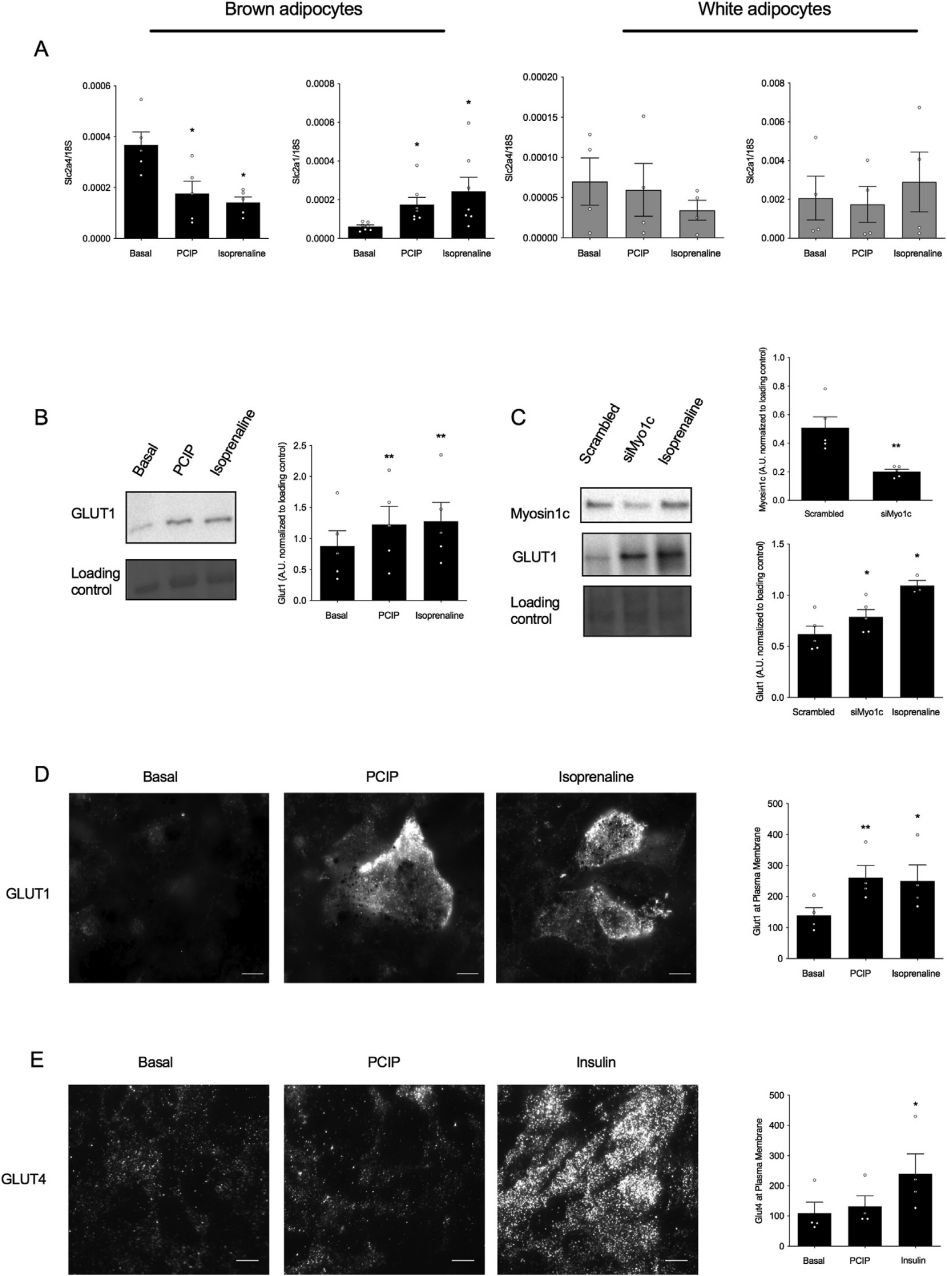
**Inhibition of myosin 1c leads to GLUT1 transcription, synthesis, and translocation in brown adipocytes**. (A) Gene expression of Slc2a1 and Slc2a4 in brown (n = 5–7) and white adipocytes (n = 4) treated with PClP (5 μM) or isoprenaline for 2 h. (B) Representative immunoblot with the relative quantification of GLUT1 protein in brown adipocytes treated with isoprenaline for 5 h (n = 5). (C) Representative immunoblot with the relative quantification of GLUT1 and myosin 1c protein in brown adipocytes treated with Myo1c siRNA (90 nM) and isoprenaline for 48 h (n = 5). (D) Representative TIRF images and their relative quantification showing GLUT1 proteins at the plasma membrane of brown adipocytes following stimulation with PClP (5 μM) or isoprenaline (1 μM) for 5 h, scale bar 10 μm (n = 4). (E) Representative TIRF images and their relative quantification showing GLUT4 proteins at the plasma membrane of brown adipocytes following stimulation with PClP (5 μM) or insulin (1 μM) for 5 h, scale bar 10 μm (n = 4). Each value represents mean ± SEM. Statistics were calculated using a paired t-test. ∗P < 0.05 and ∗∗ compared to controls.

We previously showed that β-adrenoceptor stimulation increases expression, de novo synthesis, and translocation of these newly synthesized GLUT1 transporters to the plasma membrane, increasing glucose uptake [2,4,8]. To investigate whether glucose uptake as induced through Myo1c inhibition is regulated in a similar manner, quantitative PCR was performed to measure glucose transporter gene expression in brown and white adipocytes. In accordance with earlier reports [22], the expression levels of GLUT4 at a basal state were higher in the brown adipocytes compared to the white (p = 0.0022), while GLUT1 expression was higher in the latter compared to that in the former (p = 0.0363) (Figure 2A). Treatment with PClP (5 μM) decreased GLUT4 expression in brown adipocytes but not in white adipocytes to the same extent as the positive control isoprenaline (1 μM). The inhibitory effect of adrenergic stimulation on GLUT4 gene expression in adipocytes was in accordance with earlier studies [2,23]. Contrastingly, treatment with PClP or isoprenaline for 2 h showed a significant increase in GLUT1 expression in brown adipocytes, while no such effect could be seen in white adipocytes (Figure 2A). The effects of Myo1c inhibition on the synthesis and translocation of GLUT1 was measured by the total GLUT1 content in the cell by Western blotting when brown adipocytes were stimulated with isoprenaline and PClP (Figure 2B) or exposed to siRNA targeting Myo1c for 48 h (Figure 2C) and GLUT1 at the plasma membrane by TIRF-M (Figure 2D). Cells treated with PClP for 5 h (5 μM) or siRNA for 48 h induced de novo synthesis of GLUT1 (Figure 2B–C) and GLUT1 translocation to the plasma membrane (Figure 2D). To fully exclude the role of GLUT4 translocation in PClP-mediated glucose uptake, brown adipocytes were stimulated with PClP (5 μM) for 5 h followed by quantification of GLUT4 at the plasma membrane using TIRF-M. In contrast to insulin, Myo1c inhibition did not result in GLUT4 translocation to the plasma membrane (Figure 2E). Collectively, our results suggest that glucose uptake upon Myo1c inhibition is fully dependent on GLUT1 expression, translation, and translocation.

### 2.3 Glucose uptake by myosin 1c inhibition in brown adipocytes is dependent on mTORC2 activation

**Figure 3:**
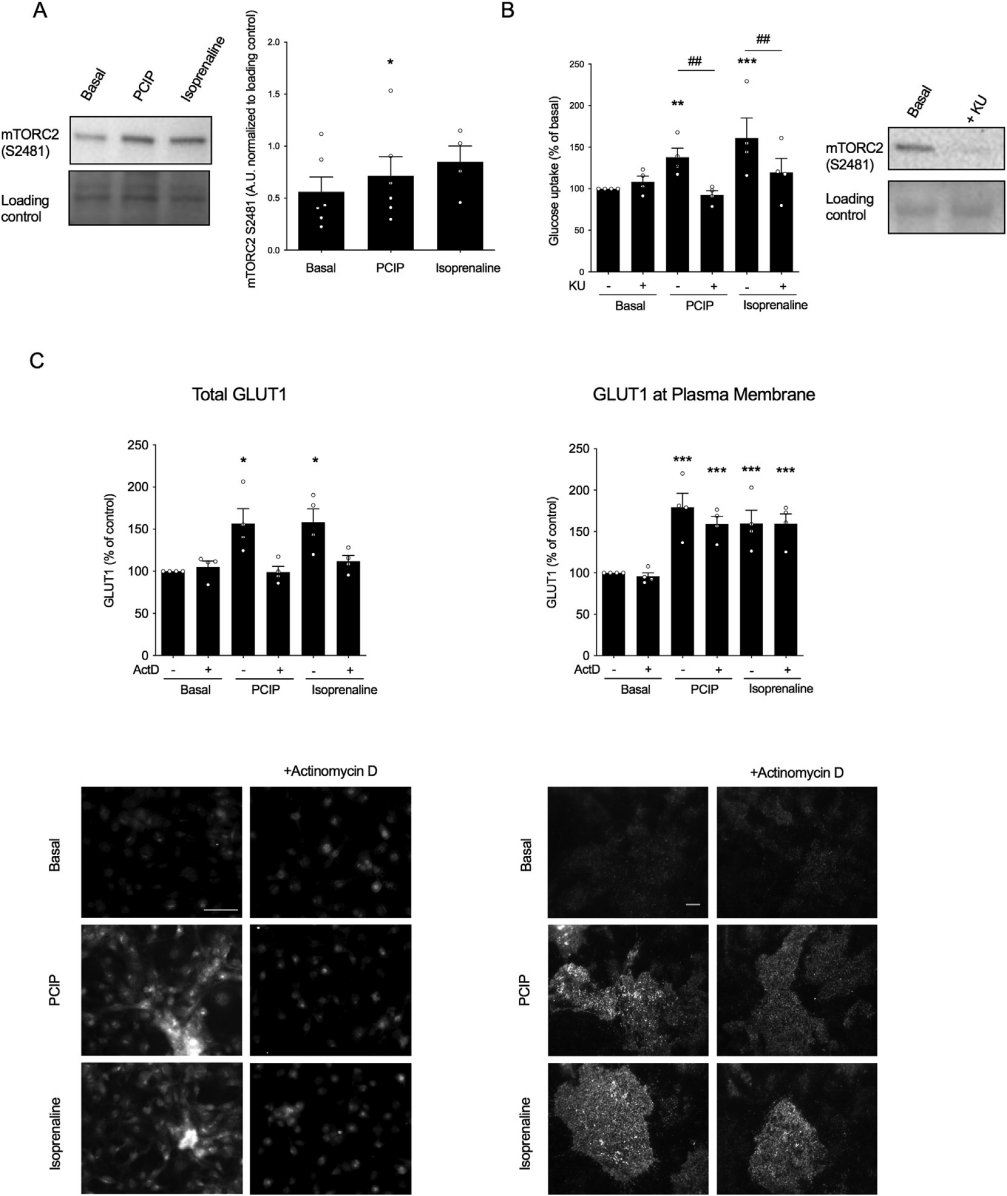
**mTORC2 phosphorylation is necessary for PClP-mediated glucose uptake in brown adipocytes**. (A) Representative immunoblot with the relative quantification of p-mTOR (S2481) in brown adipocytes treated with (5 μM) PClP or isoprenaline (1 μM) for 15 min (n = 6). (B) The effect of 5 μM of KU, an mTORC inhibitor, on PClP (5 μM) and isoprenaline (1 μM)-stimulated glucose uptake in brown adipocytes normalized to basal. Cells were pretreated with KU for 30 min prior to stimulation with either PClP or isoprenaline for 5 h (n = 4). The effect of KU pretreatment on p-mTOR (S2481) in brown adipocytes is shown as a representative immunoblot. (C) Representative epifluorescence and TIRF images and their relative quantification showing total GLUT1 and GLUT1 at the plasma membrane, respectively, for brown adipocytes stimulated with PClP (5 μM) or isoprenaline (1 μM) for 5 h in the presence or absence of 2 ug/ml of actinomycin D (n = 4), scale bars represent 50 μm and 10 μm for epifluorescence and TIRF images, respectively. Each value represents mean ± SEM. Statistics for (B) and (C) were calculated using two-way ANOVA followed by Fischer's LSD post-test. Statistics for the remaining data presented were calculated using a paired t-test. ∗P < 0.05, ∗∗P < 0.005, and ∗∗∗P < 0.0005 for differences compared to controls. ## represents statistical differences for P < 0.005 within groups.

We previously showed that β-adrenergic stimulation leads to GLUT1 translocation to the plasma membrane in brown adipocytes and that this is mediated by mTORC2 (J.M. Olsen et al., 2014). We therefore investigated the involvement of mTORC2 in this signaling event. In cells treated with PClP (5 μM) for 15 min, there was a significant increase in phosphorylation of S2481 compared to basal phosphorylation, indicative of mTORC2 activity (Figure 3A). Additionally, when cells were pretreated with mTOR inhibitor KU, both isoprenaline and PClP for 5 h failed to induce glucose uptake in brown adipocytes (Figure 3B). Collectively, these results indicate that mTORC2 is activated by Myo1c inhibition and that this activation is necessary for glucose uptake.

To further investigate if Myo1c inhibition actively translocates GLUT1 and increased GLUT1 at the plasma membrane is not a consequence of increased basal recycling due to de novo GLUT1 synthesis, brown adipocytes were treated with PClP (5 μM) or isoprenaline (1 μM) in the presence or absence of actinomycin D (2 μg/ml), a transcriptional inhibitor. Epifluorescence microscopy was performed to verify that actinomycin D indeed inhibited de novo GLUT1 synthesis (Figure 3E). In the same brown adipocyte preparations, GLUT1 was quantified at the plasma membrane using TIRF-M (Figure 3E). Both isoprenaline and PClP treatment resulted in a stimulatory effect on GLUT1 translocation, even in the presence of actinomycin D, showing that Myo1c inhibition resulted in the translocation of intracellular GLUT1 in brown adipocytes.

### 2.4 Myosin 1c inhibition induces PKA-mediated activation of CREB, p38, and ATF2 independent of an increase in cAMP

**Figure 4:**
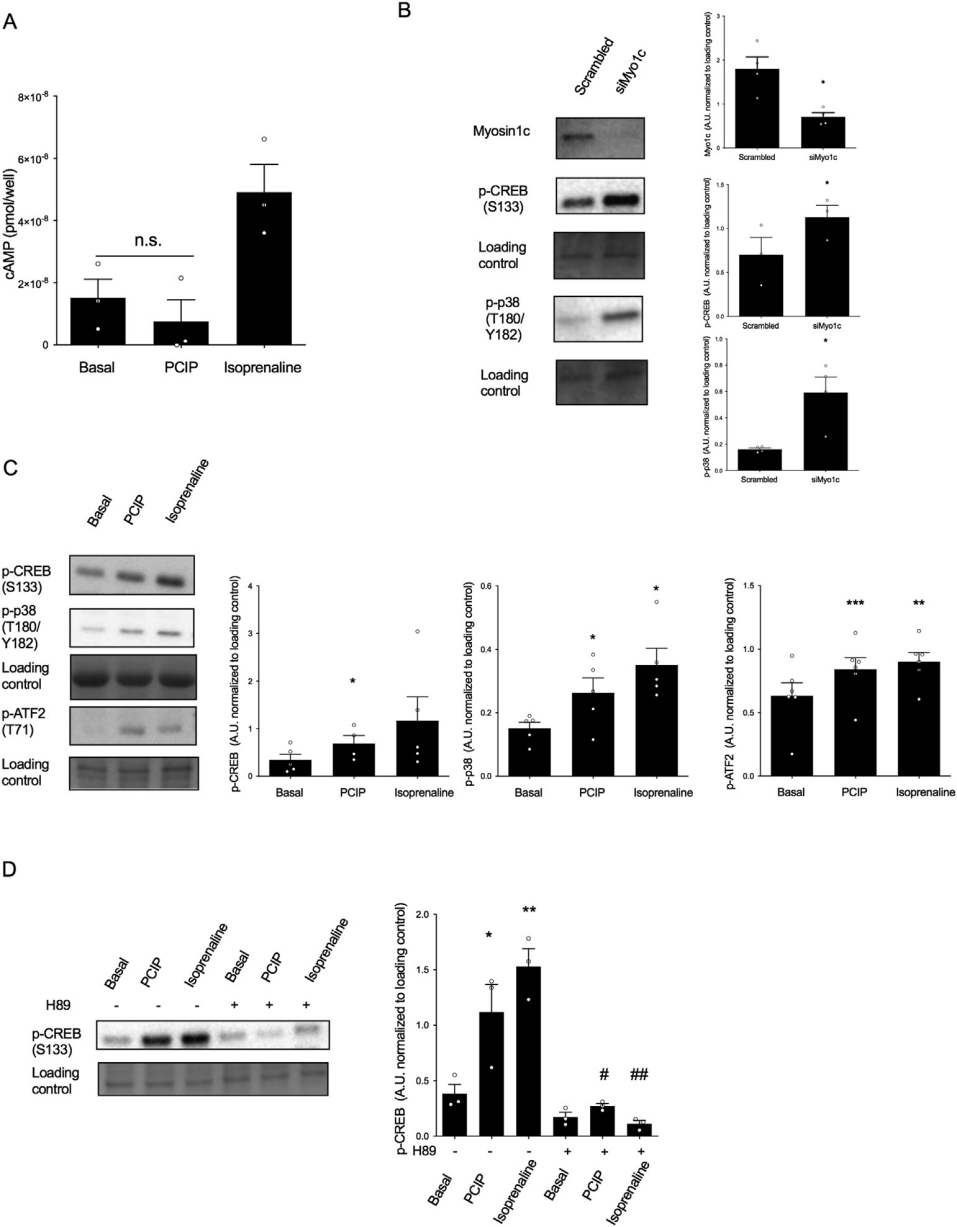
**Myosin 1c inhibition induces PKA-mediated activation of CREB, p38, and ATF2 independent of an increase in cAMP**. (A) cAMP production measured as pmol/well in brown adipocytes stimulated with PClP (5 μM) or isoprenaline (1 μM) for 15 min (n = 3). (B) Immunoblots and their relative quantifications showing the effect of Myo1c knockdown on p-CREB (S133) and p-p38 (T180/Y182) (n = 4). (C) Immunoblots and their relative quantifications showing the effect of PClP (5 μM) and isoprenaline (1 μM) stimulation on p-CREB (S133), p-p38 (T180/Y182), and p-ATF2 (T71) in brown adipocytes (n = 5–6). (D) Immunoblots and their relative quantifications showing p-CREB (S133) in brown adipocytes stimulated with either PClP (5 μM) or isoprenaline (1 μM) for 15 min in the presence or absence of H89 (40 μM), a PKA inhibitor (n = 3). Statistics for (D) were calculated using 2-way ANOVA followed by Fischer's LSD post-test. Statistics for the remaining data presented were calculated using a paired t-test. ∗P < 0.05, ∗∗P < 0.01, and ∗∗∗P < 0.001 for differences as compared to controls. # and ## represent statistical differences within groups.

As inhibition of Myo1c exerts similar effects to β-adrenoceptor agonist isoprenaline on GLUT1 transcription, translation, and translocation (Figure 2) and mTORC2 activation (Figure 3), which are all activated by cAMP in brown adipocytes, we examined the possibility that inhibition of Myo1c similarly leads to an increase in cAMP followed by the subsequent activation of PKA resulting in the downstream phosphorylation of CREB, p38, and ATF-2. The addition of isoprenaline (1 μM) increased cAMP after 15 min of stimulation. However, addition of PClP (5 μM) did not induce any production of cAMP compared to basal (Figure 4A) for up to 1 h (data not shown). However, both acute inhibition by PClP (5 μM) for 15 min or knockdown by siRNA for 48 h induced a significant phosphorylation of p-CREB and p-p38 (Figure 4B–C). Furthermore, phosphorylation of transcription factors CREB and ATF-2 by PClP was similar to isoprenaline activation (Figure 4C). Phosphorylation of CREB can involve multiple pathways, some of which are independent of both cAMP induction and PKA activation [[24], [25], [26]]. We thus further aimed to delineate the mechanism responsible for PClP-mediated phosphorylation on canonical PKA substrates. Pretreatment of brown adipocytes with the specific PKA inhibitor H-89 for 30 min completely abolished PClP induced phosphorylation on CREB, suggesting that the effects of PClP on GLUT1 transcription are primarily mediated by PKA activation.

## 3 Discussion

Myo1c has been reported to be involved in GLUT4-mediated glucose uptake in 3T3-L1 cells [15,16] and skeletal muscle [17,18]. While we reproduced inhibition of insulin-stimulated glucose uptake in primary white adipocytes, to our surprise, we discovered that removing Myo1c function in brown adipocytes and BAT resulted in the very opposite, a large increase in glucose uptake. We found that the increase in uptake was due to the synthesis and subsequent translocation of GLUT1, the primary glucose transporter important for induced glucose uptake in brown adipocytes.

### 3.1 A potent tissue-specific response

The ambient temperature initiates major adaptive changes in BAT in animals to meet the demand of thermogenesis. The proliferation and maintenance of cells are constantly regulated so that the tissue will have appropriate capacity [27], and these morphological changes need to be rigorously regulated as over- or understimulation of this potent tissue under the wrong physiological conditions could have devasting implications on survival during birth and throughout life. Upon adrenergic exposure, BAT has been shown to consume, in addition to fatty acids, high amounts of glucose both in vitro and in vivo to the extent that it can affect body glucose clearance both acutely and chronically to support lipogenesis and thermogenesis [[28], [29], [30], [31], [32], [33], [34], [35], [36], [37], [38]], with β-adrenergic stimulation increasing GLUT1 translation and translocation [8]. However, glucose uptake will not occur to the same extent under thermoneutral conditions when the need to combust additional fuel is unnecessary, although we [35] and others [39] have previously shown that β-adrenergic glucose uptake into brown adipocytes is a distinct and parallel pathway to non-shivering thermogenesis. In accordance with this, we observed weak phosphorylation on PKA substrate hormone-sensitive lipase (HSL), but we did not observe an induction of lipolysis upon PClP stimulation (data not shown) as the rate-limiting step for lipolysis is the activation of adipose triglyceride lipase (ATGL) that is not regulated directly via PKA phosphorylation [40,41]. Here we propose the hypothesis that Myo1c is an important player in this regulation of glucose uptake, impacting GLUT1 transcription in brown adipocytes by inhibiting the activation of transcription factors ATF-2 and CREB. Both p-ATF-2 and p-CREB are known to bind to CRE elements upon β-adrenergic stimulation in BAT [42] and are also present on the *Glut1* gene [43,44]. The increase in phosphorylation and binding of these transcription factors to their respective CRE-elements on the *Glut1* gene could explain the observed increase in GLUT1 protein when Myo1c is inhibited. Interestingly, Myo1c has an isoform, nuclear myosin 1 (NM1), which has been linked to nuclear functions such as DNA transcription, chromatin remodeling, and RNA maturation [[45], [46], [47], [48]]. One explanation for the increased transcriptional effects when inhibiting Myo1c could partly be due to the removal of basal repression asserted by NM1 on these CRE sites. However, a transcriptional repression function with the motor protein has not been proven in the literature and would not explain the observed induction of signaling effects and downstream transcription factor activation upon Myo1c inhibition. Activation of a PKA/CREB axis is sufficient to induce GLUT1 transcription [44]. It is therefore more plausible that the induction of this signaling pathway upon Myo1c inhibition is the primary determinant of increased GLUT1 transcription.

### 3.2 Myosin 1c as a PKA repressor

As we have shown that inhibition of Myo1c causes activation of PKA, resulting in the phosphorylation of its downstream substrates and subsequent GLUT1 transcription, we suggest that Myo1c is a repressor of basal PKA activity. In this study, we did not investigate how Myo1c interacts with PKA in this signaling event, with the important exception of ruling out increased cAMP production as the activator. This phenomenon was previously shown in several studies [49,50]. Nuclear factor κB essential modulator (NEMO)/IKK-γ is known to repress the catalytic subunit of PKA by masking its ATP-binding sites, and degradation of IKK results in the dissociation of this complex releasing the C subunit causing the activation of downstream PKA substrates independently of cAMP [51,52]. Interestingly, Myo1c has been shown to be essential for intracellular trafficking of IKK up to the plasma membrane in 3T3-L1 adipocytes [53], suggesting that our observed effects on PKA mediated by Myo1c inhibition could in part be due to disruption of IKK-PKAc complexes. There is also evidence suggesting that disassembly of certain AKAPs that contain PKA can lead to cAMP-independent activation of PKA, although the actual mechanism is not known [54]. However, it would be interesting to further investigate the potential involvement of Myo1c in the dysregulation of AKAPs, resulting in the activation of PKA substrates as inhibiting Myo1c is known to disrupt lipid raft formation [55]. PKA activation can induce MYPT1 phosphatase activity [56], a protein that is predicted to exhibit a strong interaction with Myo1c [57]. In addition, Myo1c contains a strong PKA consensus site at serine 701 (S701) [16]. Its motor function has been shown to be regulated by PKA [12]. Collectively, these could suggest the presence of a negative feedback system regulating Myo1c.

### 3.3 Motor proteins occupy isoform-specific functions in brown adipocytes

We previously shown that actin rearrangement is needed for β-adrenergic glucose uptake in brown adipocytes [4]; however, as we do not see an inhibition of glucose uptake when removing Myo1c function, it is not involved in this specific actin rearrangement function in brown adipocytes, although we cannot exclude compensatory effects from other motor proteins. Importantly, we do not achieve similar effects when removing Myo1b function, which indicates that the isoform Myo1c is particularly important in this respect. While this is the first time the function of Myo1c has been investigated in BAT, another unconventional motor protein, myosin II (MyoII), has been linked to activation of the thermogenic program through actomyosin-derived tension in BAT [58]. The authors show that actomyosin-mediated mechanics are needed for the activation of mechanosensitive transcriptional co-activators YAP and TAZ, indispensable for normal BAT function, as well as acute effects on respiration and thermogenesis. While MyoII and Myo1c have been reported to have similar functions, such as regulation of GLUT4 exocytosis in 3T3-L1 cells [15,59], in brown adipocytes, the former is required for adrenergic gene transcription, while our data suggest that the latter represses it.

### 3.4 Myosin 1c is a novel regulator of BAT glucose uptake

While we could replicate that Myo1c is required for glucose uptake in white adipocytes, an insulin-sensitive tissue that relies primarily on GLUT4 translocation for this effect, in contrast, we found that Myo1c inhibition induces glucose uptake in brown adipocytes in a GLUT1-dependent manner. We could recapitulate this effect in vivo, but could not discriminate between the contribution of de novo synthesized GLUT1 protein or the preexisting pool of GLUT1 that can be translocated upon PClP stimulus to this effect. Importantly, we did not observe this effect in white adipocytes. Although the adrenergic pathway is fully functional in white adipocytes leading to cAMP production, PKA activation, and lipolysis [41], it fails to induce glucose uptake in white adipocytes and WAT [34,60]. However, in contrast to GLUT4, the basal expression levels of GLUT1 in white adipocytes are known to be higher than in brown [22], in agreement with our data. This could be because GLUT1 is present in white adipocytes for basal uptake, which is important for the cell's storage function. While in brown adipocytes, it can be induced to enhance glucose uptake when required. Whether the Myo1c-GLUT1 axis contributes to glucose uptake in brite/beige adipocytes (white adipocytes that can be induced to acquire brown adipocyte-like features) will be the subject of future studies. Because white preadipocytes from the inguinal depot were not treated with any differentiation agents in this study, these cells were considered white [22] even if they were isolated from mice housed at room temperature at 21 °C, which can be considered as mild cold for mice. However, in a previous study, white adipocytes treated with differentiation agent rosiglitazone failed to induce the expression of GLUT1 mRNA [22], indicating that this mechanism could be specific to brown adipose tissue. In brown adipocytes, inhibition of glycolysis itself affects both basal and adrenergic thermogenesis [61], indicating the importance of the glucose substrate in these adipocytes. Furthermore, glucose tracer-based metabolomics in brown adipocytes have shown that most glucose is oxidized either through the citric acid (TCA) cycle or utilized to replenish the triglyceride pool to meet increased energy demand during short-term β_3_-AR activation [62]. We believe that brown adipocytes respond to this demand by translocating newly translated GLUT1 vesicles to rapidly sustain the induced need for substrate for thermogenesis downstream of adrenergic stimulation and that Myo1c is important in regulating this function. In conclusion, in this study, we have for the first time explored the function of Myo1c in BAT and found it regulates GLUT1 expression and translocation. Myo1c and its isoforms are therefore novel and interesting targets for research to understand cellular glucose uptake in BAT and its effect on whole animal glucose homeostasis.

## 4 Experimental procedures

### 4.1 Adipocyte precursor cell isolation

NMRI mice (3- to 4-week-old males) were purchased from Nova-SCB AB, Sweden, and housed at 21 °C. The animals were euthanized by CO_2_. Brown fat precursor cells were isolated from the intrascapular, axillary, and cervical brown adipocyte depots and white fat precursor cells were isolated from the inguinal depot as previously described [63]. The cells were filtered twice and subsequently washed. The cells were resuspended in 0.5 ml of cell culture medium per mouse dissected. All the experiments were conducted with ethical permission from the North Stockholm Animal Ethics Committee.

### 4.2 Primary cell culture of adipocytes

The cell culture medium consisted of DMEM (4.5 g d-glucose/liter) with 10% newborn calf serum (NCS), 2.4 nM of insulin, 50 UI/ml of penicillin, 50 μg/ml of streptomycin, 25 μg/ml of sodium ascorbate, and 10 nM of Hepes (Ref.: akt 40). Aliquots of 0.05 ml cell suspensions to 0.45 ml cell culture media were plated in 24-well culture dishes. The cultures were incubated in a 37 °C humidified atmosphere of 8% CO_2_ in air (Heraeus CO_2_-auto-zero B5061 incubator, Hanau, Germany). The medium was changed on days 1, 3, and 5, and the cells were used on day 7 upon assessment of sufficient differentiation by gauging lipid accumulation and transition to an adipocyte-like morphology.

### 4.3 2-Deoxy-D-[1–^3^H]-glucose uptake in primary adipocytes and in vivo

Glucose uptake was performed as previously described [64]. Cells were serum starved using a starvation medium (DMEM supplemented with 0.5% BSA, 2.4 nM of insulin, 10 nM of HEPES, 50 IU/ml of penicillin, 50 μg/ml of streptomycin, and 25 μg/ml of sodium ascorbate) the night prior to the experiment. The starvation medium was removed 30 min before and an insulin-free starvation medium was added. Inhibitors were added as indicated for 30 min before stimulation with insulin or β-adrenoceptor agonist for 2–5 h. The cells were washed with prewarmed glucose-free DMEM before glucose-free DMEM was added, and drugs and inhibitors were re-added with trace amounts of 2-deoxy-D-[1–^3^H]-glucose (50 nM) (specific activity 7.5 Ci/mmol) for 10 min. Reactions were terminated by washing in ice-cold glucose-free DMEM. The cells were lysed (400 μl of 0.2 M NaOH for 1 h at 60 °C) and the incorporated radioactivity was determined by liquid scintillation counting. For in vivo glucose uptake, male C57Bl/6 mice (12–14 weeks old) housed at 21 °C were starved for 5 h before the study and anesthetized with pentobarbital (60 mg/kg of body weight i.p.). The mice were then administered PClP (5 mg/kg i.p.) or an equal volume of vehicle (DMSO) for 2.5 h. All the experiments were conducted with ethical permission from the North Stockholm Animal Ethics Committee.

### 4.4 Fluorescence microscopy

Adipocytes were grown and differentiated on laminin-coated glass-bottomed dishes (P35GC-1.5-10-C, MatTek, Ashland, MA, USA). The cells were serum starved overnight and exposed to buffer without insulin for 30 min before they were stimulated for 2 h with drugs and immunohistochemistry was performed as previously described [65]. Epifluorescence and total internal reflection fluorescence microscope (TIRFM) images were captured using an AxioObserver D1 Laser TIRF 3 system (Carl Zeiss) equipped with an alpha Plan-Apochromat 63 × /1.46 oil objective lens, Plan-Apochromat 20x/0.6 objective lens, diode-pumped solid-state (561 nm/20 mW) laser, 86HE (561 nm) shift-free filter set, and filter set 49 for DAPI. Images were captured with an AxioCam MRm Rev 3 camera (Carl Zeiss), and the system was controlled by ZEN Blue 2011 software. The penetration depth of the evanescent field was estimated to be 160 nm. Images were acquired with a readout rate of an average exposure time of 500 ms. The intensities of all the regions of interest (ROIs) were normalized to the controls with 5 ROIs per image with at least 5 images per condition.

### 4.5 Immunoblotting

Adipocytes were grown and differentiated in 48-well plates and serum starved the night prior to the experiment. On day 7, the cells were challenged with PClP or isoproterenol as indicated and harvested as previously described [65]. Immunoblotting was performed as previously described [66]. The primary antibodies p-CREB, p-p38, and pATF2 (1:1000) were from Cell Signaling (Danvers, MA, USA), GLUT1 (1:1000) was from Abcam, and myosin 1c (1:1000) was from Santa Cruz Biotechnology. All the primary antibodies were detected using a secondary antibody (horseradish peroxidase-linked anti-rabbit IgG or anti-muse IgG, Cell Signaling) diluted to 1:2000.

### 4.6 Antibody transfection

Primary brown adipocytes differentiated in 48-well plates were transfected on day 7 with 0.5 ug/well of Myo1c (sc-136,544), Myo1b (sc-393,053), and IgG (OZ Biosciences) antibodies in Ab-DeliverIN (OZ Biosciences) reagent dissolved in serum-free medium for 8–10 h 2DG uptake was measured, isoprenaline was used as a positive control for the induction of glucose uptake, and IgG antibody was delivered into the cells to account for any non-specific induction of glucose uptake.

### 4.7 SiRNA-mediated knockdown

Primary brown adipocytes were transfected with Myo1c siRNA as previously described [67]. Briefly, scrambled (sc-37007) or Myo1c siRNA (sc-44614) purchased from SCBT was delivered into brown adipocytes on days 5–6 using Lipofectamine RNAiMAX (Thermo Fisher Scientific) dissolved in Opti-MEM (Gibco) at a final concentration of 90 nM. After 48 h, the cells were harvested for immunoblotting.

### 4.8 Gene expression analysis

Cells were seeded in 12-well plates on day 7 and stimulated with PClP (5 μM) or isoprenaline stimulation (1 μM) for 2 h. The cells were harvested with TRI reagent and the RNA was isolated. Then 500 ng of RNA was reverse transcribed to cDNA with a High-Capacity cDNA Reverse Transcription Kit (Thermo Fisher Scientific) and qPCR was performed using SYBR Green JumpStart Taq ReadyMix (Sigma). The following protocol was used: 50 °C for 2 min, 95 °C for 10 min, 39 cycles at 95 °C for 15 s and 60 °C for 1 min, 65 °C for 5 s, and 95 °C for 0.5 s. The gene expression was established using the 2-Ct value and normalized to 18S.

**Table:** 

Target	Forward primer 5′-3′	Reverse primer
18S	AGTCCCTGCCCTTTGTACACA	CGATCCGAGGGCCTCACTA
Slc2a1	GATCCCAGCAGCAAGAAGG	TGACACCAGTGTTATAGCCG
Slc2a4	GCTGTATTCTCAGCTGTGCT	TTCAATCACCTTCTGTGGGG

### 4.9 cAMP assay

To measure cAMP production, a competition assay AlphaScreen cAMP Assay Kit with 1,000 assay points (PerkinElmer) was used (buffers were produced and the procedure followed the manufacturer's protocol). The cells were placed in stimulation buffer with IBMX and exposed to PClP (5 μM) or isoprenaline (1 μM) stimulation for 15 min.

## 5 Statistics

Statistical analysis was performed using Prism 9.0. Tests used are described in the figure legends. Each adipocyte preparation consisted of pooled tissues from 4 to 6 mice and considered one data point. The brown and white preadipocytes were not isolated from the same animals.
